# Hypothalamic Atrophy and Textural Changes in Polyglutamine Ataxias

**DOI:** 10.1007/s12311-026-01978-4

**Published:** 2026-03-23

**Authors:** Livia Rodrigues, Thiago J. R. Rezende, Alberto R. M. Martinez, Breno Massuyama, Jose Luiz Pedroso, Orlando G. P. Barsottini, Juan Eugenio Iglesias, Simone Appenzeller, Letícia Rittner, Marcondes C. França

**Affiliations:** 1 Department of Neurology, School of Medical Sciences, University of Campinas (UNICAMP), Campinas, SP Brazil; 2 School of Medical Sciences, Brazilian Institute of Neuroscience and Neurotechnology (BRAINN), University of Campinas (UNICAMP), Campinas, SP Brazil; 3 Department of Neurology, Federal University of São Paulo (UNIFESP), São Paulo, SP Brazil; 4 Department of Computer Engineering and Automation, School of Electrical and Computer Engineering, University of Campinas (UNICAMP), Campinas, SP Brazil; 5 School of Medical Sciences, University of Campinas (UNICAMP), Campinas, SP Brazil; 6 Massachusetts General Hospital, Harvard Medical School, Boston, USA; 7 Centre for Medical Image Computing, University College London, London, UK; 8 Computer Science and Artificial Intelligence Laboratory, Massachusetts Institute of Technology, Boston, USA; 9 Department of Neurology, University of Campinas (UNICAMP), Rua Tessália Vieira de Camargo, 126. Cidade Universitária “Zeferino Vaz”, Campinas, SP 13083-887 Brazil

**Keywords:** Ataxia, Polyglutamine disease, Hypothalamus, MRI, Volumetry

## Abstract

Background. Spinocerebellar ataxia (SCA) presents a complex genetic landscape, with over 40 subtypes. These autosomal dominant disorders manifest late onset and severe disability, primarily impacting the cerebellum but also involving other nervous system structures. While a single study has linked hypothalamic atrophy to SCA3, further research is needed to confirm and/or expand this association. This study aimed to investigate hypothalamic involvement in PolyQ SCAs, focusing on SCA 1, 2, 3, and 6. Methods. We studied 135 adult patients with genetically confirmed SCA (SCA1, SCA2, SCA3, and SCA6) and 117 healthy controls. Hypothalamic subregions were segmented using H-SynEx, and both volumetric and texture features were extracted from MR images. Group differences were assessed with the Mann–Whitney U test, and associations with disease duration and severity (measured by SARA) were examined using Spearman’s rank correlation. Results. Significant atrophy of hypothalamic subregions was observed in SCA1, SCA3, and SCA6 when compared with controls, with the anterior subregion being affected in all cases. Texture analysis revealed widespread alterations in SCA1, SCA3, and SCA6 across all subregions. In SCA1 and SCA3, both volumetric and texture measures correlated with disease duration and SARA scores. These findings suggest that hypothalamic involvement in SCAs is complex and may occur before overt clinical manifestations. Key words: Ataxia, Polyglutamine disease, Hypothalamus, MRI, Volumetry

## Introduction

Spinocerebellar ataxia (SCA) is an autosomal dominant degenerative disorder with remarkable phenotypic and genotypic heterogeneity. This is typically a late-onset and very disabling condition. The unifying feature in all SCAs is the presence of slowly progressive cerebellar ataxia either in isolation or combined with other neurological manifestations [12]. To date, there are more than 40 genetic subtypes of SCAs; these are named sequentially in the chronological order of loci discovery (from SCA1 up to SCA50) [4]. The overall prevalence of SCAs ranges from 1 to 5 per 100,000, but some relevant regional variations exist [5]. The most common subtypes worldwide are SCA1, 2, 3 and 6 [23]. These subtypes share a common genetic mechanism – they are all caused by unstable (CAG) repeat expansions within coding regions of specific genes. This leads to the production of aberrant proteins with abnormally long polyglutamine chains (PolyQ diseases). The ultimate consequence is the formation of intracellular protein aggregates and ensuing neuronal death [14].

The cerebellum is the main target of neurodegeneration in SCAs, but other central as well as peripheral nervous system structures are affected as well. There is now pathological and imaging evidence of basal ganglia, brainstem and spinal cord involvement in many specific SCAs [13, 19, 20, 24]. Such extra-cerebellar damage is particularly relevant for SCA 1, 2 and 3. In these PolyQ ataxias, some clinical manifestations are indeed related to basal ganglia (e.g., dystonia in SCA3) and spinal cord (e.g., sensory loss in SCA 1, 2 and 3) impairment, rather than cerebellar involvement per se. The hypothalamus is a small but extremely important brain region located deep in the ventral part of the diencephalon. It plays a key role in endocrine and autonomic control and is also part of the limbic system [25]. Hypothalamic abnormalities have been consistently shown in vivo in many degenerative diseases lately, such as amyotrophic lateral sclerosis and frontotemporal dementia [9, 26]. In these studies, authors relied upon high-resolution brain MRI scans and novel segmentation pipelines to compute hypothalamic volumes [9, 26]. So far, little is known about hypothalamic involvement in SCAs. There is a single study published by Guo et al., [10] who reported hypothalamic atrophy as well as positive correlation between atrophy and body mass index in Chinese patients with SCA3 [10]. It is not yet clear whether the same findings hold true for other PolyQ SCAs. In addition, no information is available on the pattern of atrophy or textural abnormalities within hypothalamic subregions.

Herein, we attempted to address these open questions regarding hypothalamic involvement in PolyQ SCAs. We hypothesize that the hypothalamus is morphologically abnormal in SCA 1, 2 and 3, but not in SCA6, since this is known to be a pure cerebellar ataxia with no anatomical damage outside the cerebellum [24]. To test that, we employed a novel and validated deep learning-based pipeline to compute hypothalamic morphometry (global and subregions) as well as texture analysis in a large cohort of subjects with molecular confirmation of SCA 1, 2, 3 and 6 [21]. Results were then compared with matched controls and assessed for possible correlations with genotypic/phenotypic data.

## Methods

### Subject Selection and Clinical Evaluation

The study included 135 adult symptomatic patients with molecularly confirmed polyglutamine SCA: 31 with SCA1, 12 with SCA2, 92 with SCA3, and 17 with SCA6, all regularly followed at the University of Campinas Hospital.

To ensure age- and sex-matching, two groups of healthy subjects were selected as controls: one for SCA1, SCA2, and SCA3, and another for SCA6, as the SCA6 cohort was considerably older than the others. The first control group comprised 100 subjects, and the second, 17 subjects. Age was compared using independent samples t-tests, and sex distribution was compared using Fisher’s exact tests. Each SCA group was compared with its corresponding control group (SCA1–3 vs. Controls for SCA1–3 and SCA6 vs. SCA6 Controls). Control participants were carefully screened to exclude any history of neurological, psychiatric, or systemic disorders, including endocrine or metabolic conditions that could be associated with hypothalamic dysfunction (e.g., diabetes insipidus, thyroid or adrenal disorders, hypogonadism, or sleep–wake disturbances). None of the control individuals had known neuroendocrine disorders or conditions affecting hypothalamic function.

For each patient, demographic (age, sex) and clinico-genetic data (disease duration, expanded CAG repeat length, neurological findings) were collected. Basic demographic and clinical characteristics of all enrolled subjects are summarized in Table 1. By design, there were no significant differences in age or sex distribution between patients and their respective control cohorts.

**Table 1:** Demographic, clinical and genetic data of the ataxic study participants

Group	*n* (male/female)	Age (mean ± std)	CAG repeat length, long allele	Mean SARA	Mean time from ataxia onset
SCA1	31 (21 M/10F)	44.55 ± 8.63	44.6 ± 4.17	15 ± 6.75	8 ± 6.58
SCA2	12 (6 M/6F)	40.5 ± 20.22	45.88 ± 10.53	23.25 ± 11.38	9.6 ± 5.95
SCA3	92 (40 M/52F)	48.33 ± 12.2	71.98 ± 3.71	13.35 ± 7.9	13.39 ± 7.15
SCA6	17 (10 M/7F)	67.88 ± 7.88	22.17 ± 1.27	13.94 ± 6.34	14.13 ± 6.97
Control (SCA6)	17 (8 M/9F)	65.35 ± 3.22	-	-	-
Control (SCA1, SCA2 and SCA3)	100 (50 M/50F)	47.29 ± 8.63	-	-	-

### MRI Acquisition

All participants underwent an MRI scan utilizing a 3T Philips Achieva scanner (Philips, Best, The Netherlands) with standard 8-channel head coils. High-resolution 3D T1 volumetric images of the brain in sagittal orientation were acquired, featuring a voxel matrix of 240 × 240 × 180, a voxel size of 1 × 1 × 1 mm^3, a TR/TE of 7/3.201 ms, and a flip angle of 8°.

### Hypothalamic Segmentation

The image processing procedures were executed using H-SynEx [21], an automated segmentation method designed for delineating the hypothalamus and its respective subregions. This method was developed using ultra-high-resolution ex vivo images applied to deep learning models and works for different in vivo MRI sequences and resolutions. The output of this method encompasses the segmentation of 10 distinct hypothalamic subregions, namely right and left anterior-inferior, anterior-superior, tuberal-inferior, tuberal-superior, and posterior.

The segmentation protocol is defined in [21]. In summary, the superior boundary was defined using the recess located dorsal to the hypothalamus. Ventrally, it is delineated by the optic tract and the hypothalamic sulcus in the most rostral slices. Additionally, the most anterior coronal slice is determined by the visibility of the anterior commissure, while the most caudal coronal slice is identified by the first slice where mammillary bodies are no longer visible. The segmentation includes the mammillary bodies but excludes the fornix and optic tract.

For subregion delineation, the most rostral slice of the anterior subregion is the most rostral slice of the hypothalamus in coronal view. The anterior region’s most caudal part is defined by the first slice where the anterior commissure is visible from the sagittal view. The tuberal region begins posteriorly to the coronal slice where the anterior regions are visible and their most rostral slice is the one before the mammillary bodies are visible. The posterior subregion is defined by the mammillary bodies. Superior and inferior divisions are delineated by a horizontal line on the coronal slice connecting the most medial to the most lateral point of the hypothalamus.

SynthSeg [2], an advanced deep learning-based approach specifically designed for whole-brain segmentation, was used to find the total intracranial volume (TIV) of each subject in this study in order to remove head size effects of the analysis.

### Analyzed Hypothalamic Attributes

The analyses conducted here are based on the hypothalamus subregions volumes and texture features extracted from the histogram of MRI intensities in the voxels labeled as hypothalamus by H-SynEx.

#### Texture

The MR images contain volumetric elements known as voxels. Each voxel is characterized by a numerical value representing its grayscale intensity. By examining the distribution of voxel values within a region of interest, the texture of that region is analyzed. Despite T1w MR imaging does not provide microscopic-level information, certain pathological conditions may induce histological alterations that manifest as changes in grayscale patterns within the T1w MR images of the affected area. Such alterations can be discerned through texture analysis. In fact, one can find in the literature numerous studies investigating texture characteristics in MR images across different neurological disorders. Here, we applied a statistical texture analysis employing histogram-based parameters [3].

The histogram of an image is a function that records the frequency of occurrence of different grayscale levels within the image. From the histogram, various attributes can be extracted. The mean provides the average grayscale value of the image. Variance quantifies the deviation of gray pixel values from the mean. Skewness examines the symmetry of the histogram, determining whether there is a prevalence of light or dark pixels compared to the mean. A positive skewness implies an abundance of pixels with values below the mean, whereas a negative skewness indicates an abundance of pixels with values above the mean. Lastly, kurtosis measures the uniformity of pixel distribution relative to a normal distribution. Negative values denote a flat distribution, whereas positive values indicate a peaked distribution. Finally, the entropy measures the randomness of the gray level distribution [15, 17].

Before performing the analysis, all images underwent bias field correction and gray-level normalization to the mean white matter value.

#### Volumetry

Meanwhile, the volumetric analysis aims to assess variations in subregion volumes between patient and control groups. For this analysis, all subregion volumes are initially normalized by the TIV of each subject by division, ensuring a more accurate comparison by accounting for individual variation in overall brain size.

### Experiments Conducted

#### Differences in Hypothalamic Attributes Between SCA Patients and Healthy Controls

To determine whether SCA patients exhibit differences in hypothalamic attributes compared to healthy controls (HCs), we performed group-level analyses. A Mann–Whitney U test was applied with a 95% confidence level. Multiple comparisons across hypothalamic subregions were controlled using Bonferroni correction (significance threshold: *p* < 0.05).

#### Associations of Hypothalamic Attributes with Disease Duration and Severity

We next evaluated whether hypothalamic attributes were associated with disease duration and severity. Disease severity was assessed using the Scale for the Assessment and Rating of Ataxia (SARA) [22], administered on the same day as MRI acquisition by board-certified neurologists. Spearman’s rank correlation coefficient was used to assess the strength and direction of associations. Multiple comparisons across subregions were corrected using the Bonferroni method (significance threshold: *p* < 0.05).

### Statistical Analysis

All statistical analyses were non-parametric and conducted using the Python library Scipy.

### Ethics Approval

The data used for this study received approval from the local ethical committee (CEP/Conep, number 3435027) and underwent complete anonymization. All participants were adequately informed and provided their consent by signing a consent form to participate in the study.

### Data Availability

The principal author (Livia Rodrigues) has full access to the data used in the analyses presented in this manuscript. Anonymized data not published within this article will be made available by request from any qualified investigator.

## Results

### Study Cohorts

Detailed demographic and clinical data of each cohort is shown in Table 1. Regarding demographics, no significant differences relative to controls were observed for age (SCA1: *p* = 0.49; SCA2: *p* = 0.10; SCA3: *p* = 0.72; SCA6: *p* = 0.23) or sex distribution (SCA1: *p* = 0.10; SCA2: *p* = 1.00; SCA3: *p* = 0.39; SCA6: *p* = 0.73), confirming appropriate demographic matching between patients and controls.

### Differences in Hypothalamic Attributes Between SCA Patients and Healthy Controls

**Fig. 1:**
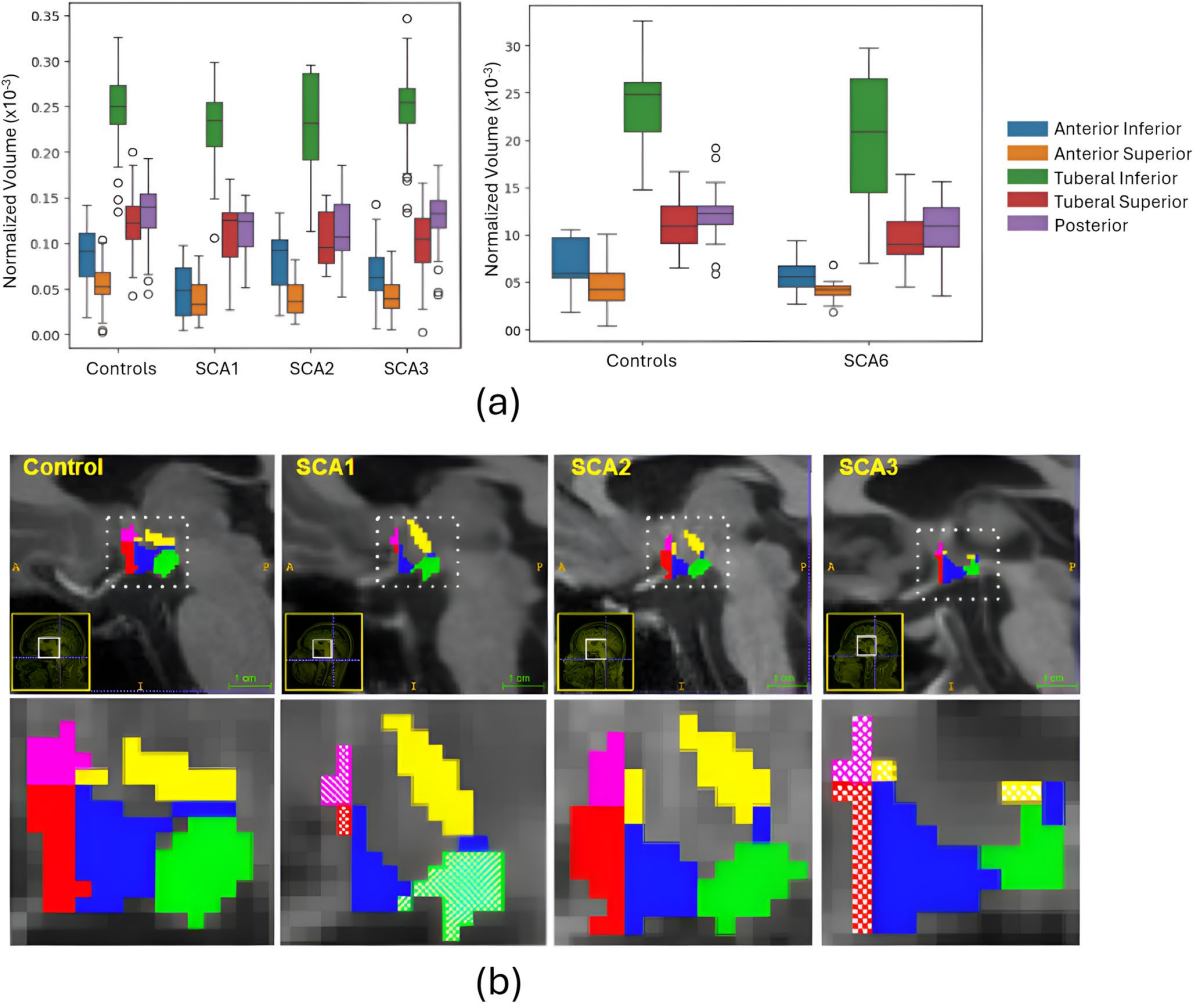
(**a**) Boxplot showing normalized volumes of hypothalamic subregions in patients and matched controls. Left: SCA1, SCA2 and SCA3 cohorts. Right: SCA6 cohort (**b**) Sagittal view of hypothalamus segmentation across four different female subjects with similar age (between 51 and 53 years). Each color represents a different subregion: Pink (anterior superior), red (anterior inferior), yellow (tuberal superior), blue (tuberal inferior) and green (posterior). Dashed regions on SCA1 and SCA3 represent the subregions where significant differences were found

We observed volumetric reductions in several hypothalamic subregions in SCA1, SCA3, and SCA6 (Fig. 1; Table 2). Statistically significant reductions were found in the inferior and superior anterior subregions for these groups. Additionally, significant volume loss was detected in the tuberal superior subregion in SCA3 and SCA6 and in the posterior subregion in SCA1 and SCA6.

**Table 2:** Normalized volume and texture attributes average values across different groups and hypothalamus subregions

Atribute	Subregions	Controls (SCA1, SCA2, SCA3)	Controls (SCA6)	SCA1	SCA2	SCA3	SCA6
Normalized Volume (*x* 10^− 5^)	ant_inf	8.75	6.77	4.79**	8.20	6.55**	5.51**
	ant_sup	5.49	4.62	3.81**	4.06	4.16**	4.10**
	tub_inf	25.03	23.83	22.85	23.01	24.95	20.65
	tub_sup	12.22	11.13	11.03	10.45	10.26**	9.74*
	posterior	13.45	12.25	11.47**	11.62	13.09	10.25**
Texture (Mean)	ant_inf	203.07	200.76	201.65	205.27	201.52	302.38**
	ant_sup	217.80	191.74	204.66*	201.62	206.10**	310.84**
	tub_inf	213.34	211.12	201.54	205.98	202.98*	296.04**
	tub_sup	231.80	205.59	203.77**	206.73*	201.86**	295.97**
	posterior	203.39	210.81	211.45	217.88	215.28	323.91**
Texture (Variance)	ant_inf	3268.08	6454.12	666.99**	858.36**	691.12**	3339.20
	ant_sup	5167.88	5573.22	552.89**	710.36**	618.25**	2720.87*
	tub_inf	6702.49	7707.37	835.20**	915.79**	895.22**	5356.33
	tub_sup	6054.17	7572.04	885.99**	988.39**	881.93**	6789.90
	posterior	5620.86	6928.78	889.13**	949.30**	887.91**	5610.033
Texture (Skewness)	ant_inf	-0.03	0.21	0.29	0.32	0.44**	-0.75*
	ant_sup	-0.25	0.06	-0.57	-0.64	-0.35	-1.22**
	tub_inf	-0.11	-0.08	-0.04	-0.06	-0.01	-0.80
	tub_sup	-0.51	0.14	-0.39	-0.29	-0.32*	-0.71
	posterior	-0.13	-0.11	-0.66	-0.40	-0.53	-1.03**
Texture (Kurtosis)	ant_inf	0.78	0.23	0.61	1.52	1.07**	1.53**
	ant_sup	1.03	-0.11	0.51	0.55	0.50	1.45**
	tub_inf	1.06	0.44	-0.05	0.33	0.11	1.21
	tub_sup	0.73	2.03	-0.11	-0.05	-0.20	0.11
	posterior	0.91	-0.14	-0.19	-0.03	0.11	0.78*
Texture (Entropy)	ant_inf	1.74	1.76	1.41**	1.53**	1.49**	1.53**
	ant_sup	1.60	1.64	1.37**	1.39**	1.42**	1.45**
	tub_inf	1.88	1.90	1.72**	1.71**	1.74**	1.79*
	tub_sup	1.78	1.80	1.67**	1.65**	1.64**	1.72
	posterior	1.77	1.80	1.66**	1.65**	1.68**	1.73

*p-value<0.05 **p-value<0.01 when comparing patient and control groups for Mann-Whitney test (using Bonferroni correction for five subregions)

Texture analysis revealed significant findings in variance and entropy across all subregions for SCA1, SCA2, and SCA3 in all cases (Table 2). For SCA6, significant findings were noted for variance in the anterior superior subregion and for entropy in both the anterior and tuberal inferior subregions. Skewness and kurtosis did not show significant differences in any subregion for SCA1 and SCA2. In SCA3, significant findings for skewness and kurtosis were observed only in the anterior inferior subregion. For SCA6, significant differences in skewness and kurtosis were found in both anterior and posterior subregions.

Additionally, significant differences in the mean were observed in all subregions only for SCA6. For SCA1, significant differences in the mean were found in the anterior superior and tuberal superior subregions; for SCA2, in the tuberal superior subregion; and for SCA3, in the anterior superior and both tuberal subregions. Notably, the mean values for the patient group were lower than those of the control group in SCA1, SCA2, and SCA3, whereas in SCA6, the patient group had higher mean values compared to the control group.

### Associations of Hypothalamic Attributes with Disease Duration and Severity

Significant correlations were observed between hypothalamic imaging texture parameters and disease severity (expressed using SARA scores) for SCA1, SCA2, and SCA3 (Table 3). In SCA1, a moderate inverse correlation was found between volume and severity in the tuberal superior and posterior subregions, suggesting that these regions exhibit greater atrophy in more severe cases. A moderate negative correlation was also observed between tuberal superior volume and disease duration. Additionally, significant correlations were identified between textural attributes and the posterior subregion.

**Table 3:** Correlation between SARA/duration and volume/texture attributes

		SCA1	SCA2	SCA3
Volume	SARA	Tuberal Superior (*r*=-0.59) Posterior (*r*=-0.50)	-	Anterior Inferior(*r* = -32) Tuberal Inferior (*r*=-0.30) Posterior (*r*=-0.47)
	Duration	Tuberal Superior (*r*=-0.58)	-	Posterior (*r* = -0.30)
Texture (Mean)	SARA	-	-	Anterior Superior (*r*=-0.29) Posterior (*r*=-0.48)
	Duration	Posterior (*r* = -0.47)	-	Posterior (*r*=-0.28)
Texture (Variance)	SARA	-	Posterior(*r* = 0.73)	Posterior (*r* = 0.3)
	Duration	-	-	Posterior (*r* = 0.34)
Texture (Kurtosis)	SARA	-	-	Anterior Inferior (*r*=-0.31) Tuberal Superior (*r*=-0.29) Posterior (*r*=-0.55)
	Duration	Posterior (*r* = -0.46)	-	Posterior (*r*=-0.33)

r represents Spearman *r* for a corrected *p*-val < 0.05. Interpretation of Spearman’s r coefficient applied to medicine - analysis for absolute values [1]: 0:None; 0.1–0.29:poor; 0.3–0.59:Fair; 0.6–0.79: Moderate; 0.8–0.99: Very Strong; 1: Perfect

In SCA3, we found a fair negative correlation between the anterior inferior, tuberal inferior, and posterior subregions and disease severity. A fair negative correlation was also observed between the posterior subregion and disease duration. Additionally, fair to moderate correlations were identified between texture features of several subregions and both disease severity and duration. Notably, the posterior subregion showed correlations between all texture attributes and volume when analyzing both duration and severity.

Finally, in SCA2, we could find a strong correlation between the Posterior subregion variance and disease severity.

No significant correlations were found for SCA6.

## Discussion

The main contribution of this study is the assessment of hypothalamic damage in vivo in patients with SCAs. Even though there is a previous imaging-based report of hypothalamic atrophy in SCA3 [10], we were able to take a step forward in the current study. Indeed, additional SCA subtypes were investigated (SCA1, SCA2, and SCA6), enabling comparisons between each of these groups and controls. Furthermore, a novel and comprehensive imaging pipeline was employed, which assesses not only volumetry but also texture attributes in all hypothalamic subregions [21]. Our results indicate that hypothalamic abnormalities are a frequent counterpart in polyglutamine SCAs. However, the pattern seems to be disease-specific. SCA1 and SCA3 cohorts presented severe and widespread volumetric changes. Although SCA6 was initially expected to show no hypothalamic involvement, significant volumetric and texture abnormalities were also detected in this group, albeit with a pattern that differed from SCA1 and SCA3. In contrast, the SCA2 cohort showed largely preserved hypothalamic morphometry. Such differences across genotypes admit 2 possible explanations. The patterns of hypothalamic damage may be truly different across SCAs. It is possible that neurodegeneration extends to the hypothalamus in some SCAs, but not others. This hypothesis agrees with the phenotypic differences across genotypes. Hypothalamus-related manifestations such as dysautonomia and sleep disorders, are indeed common in some (SCA1 and 3), but not all (SCA6). Alternatively, this phenomenon may simply reflect the uneven sample sizes across comparison groups, which may have turned some SCA cohorts underpowered to detect differences relative to controls. Additional studies with larger SCA sample sizes will be important to clarify this point.

Another important finding was the mismatch between volumetry and texture analyses. There were statistically significant results in texture analyses, even within subregions where atrophy was not apparent (and the opposite was rather unusual). The dissociation between texture abnormalities and preserved hypothalamic volumes, particularly in SCA6, supports the hypothesis that texture changes may represent an earlier or more sensitive marker of hypothalamic involvement. This interpretation, however, should be validated in longitudinal studies.

Most pathological reports on SCA1 and SCA3 focus on cerebellum, basal ganglia, and brainstem tissue changes. So far, no neuropathologic study has attempted to characterize hypothalamic abnormalities in these diseases. Despite that, we hypothesize that neuronal loss and gliosis are the probable tissue counterparts of the hypothalamic imaging abnormalities reported here. Gene expression and neurochemical data are in line with this assumption. Indeed, both *ATXN1* and *ATXN3* are highly expressed in the hypothalamus, suggesting that this structure may be vulnerable to the deleterious effects of mutant ATXN1/ATXN3 expression (GTEx Portal [7, 8]). Arginine vasopressin (AVP) is a hormone relevant for body fluid homeostasis produced by hypothalamic neurons and secreted by the pituitary gland [27]. Jiang et al., found reduced hypothalamic concentration of AVP in transgenic mice with SCA3 relative to control littermates [11], suggesting hypothalamic damage/dysfunction takes place in the disease.

Correlation analyses between imaging parameters and clinical data give us some insights into the nature of SCA-related hypothalamic damage. In SCA1 and SCA3, hypothalamic volumes were negatively correlated with disease duration and severity. This suggests that hypothalamic changes progress over the disease course rather than remaining static. Although intuitive, this hypothesis needs to be validated in longitudinal studies. There are many potential phenotypic correlates for the hypothalamic morphometric changes herein reported. In SCA1, SCA3, and SCA6, volumetric abnormalities were most noticeable at the anterior hypothalamus, a region known to be involved with circadian rhythm and autonomic control. It is thus possible that anterior hypothalamic damage may account at least in part for sleep and/or autonomic disorders, which are common in both SCA subtypes [16, 18, 28]. The SCA3 cohort also presented superior tuberal volumetric reduction relative to controls. This specific region plays an important role in the control of feeding behavior and metabolic homeostasis. So, one may assume such regional damage can contribute to the wasting syndrome noticed in many subjects with SCA3 (sometimes found well before dysphagia appears) [6]. This assumption is in line with Guo et al., who found tuberal volumetric reduction as well as correlation between tuberal volume and body mass index in Chinese patients with SCA3 [10].

Despite the original contributions, this study has some noteworthy limitations. The sample sizes of the SCA2 and SCA6 cohorts are relatively small and may have been underpowered to detect mild abnormalities relative to controls. In addition, clinical phenotyping of the enrolled patients did not include nutritional (e.g., body mass index) or autonomic assessments paired to MRI acquisition. This would have been important to fully appreciate the clinico-biological correlates of hypothalamic damage in each SCA subtype. Finally, we cannot establish direct causal relationships based on the correlation analyses herein reported (e.g., between hypothalamic attributes and ataxia severity). At this point, it becomes clear that additional studies are needed to better understand hypothalamic involvement in SCAs. These should include larger and well characterized cohorts in a longitudinal setting. Considering SCAs are rare (some subtypes are indeed ultra rare) disorders, this is a challenging task that will require multicentric initiatives - such as the ENIGMA-ataxia consortium - to be accomplished (Rezende et al., 2023).

In conclusion, we have shown that quantitative neuroimaging (morphometry and texture analyses) is able to detect hypothalamic damage in vivo in polyglutamine SCAs. The pattern of hypothalamic abnormalities is distinct across SCA subtypes, with severe and widespread changes in SCA1 and SCA3, largely preserved morphology in SCA2, and a distinct pattern of volumetric and texture alterations in SCA6. Furthermore, there is an antero-posterior gradient of damage in SCA1 and SCA3 (more severe in anterior regions of the hypothalamus). The clinical and biological correlates of such damage still deserve further investigation. 

## Data Availability

The principal author (Livia Rodrigues) has full access to the data used in the analyses presented in this manuscript. Anonymized data not published within this article will be made available by request from any qualified investigator.

## Glossary

SCA: Spinocerebellar Ataxia.
PolyQ: Polyglutamine.
MRI: Magnetic Resonance Imaging.
TIV: Total Intracranial Volume.
SARA: Scale for the Assessment and Rating of Ataxia.
